# Concrete for Precast Blocks: Binary and Ternary Combination of Sewage Sludge Ash with Diverse Mineral Residue

**DOI:** 10.3390/ma13204634

**Published:** 2020-10-17

**Authors:** Francisco Baeza-Brotons, Jordi Payá, Oscar Galao, Marcos G. Alberti, Pedro Garcés

**Affiliations:** 1 Department of Civil Engineering, Universitat d’Alacant, Carretera San Vicente del Raspeig S/N, San Vicente del Raspeig, 03690 Alicante, Spain; fbaeza.brotons@ua.es (F.B.-B.); oscar.galao@ua.es (O.G.); pedro.garces@ua.es (P.G.); 2 Institute of Concrete Science and Technology (ICITECH), Universitat Politècnica de València, Camino de Vera S/N, 46022 València, Spain; jjpaya@cst.upv.es; 3 Departamento de Ingeniería Civil: Construcción, E.T.S. de Ingenieros de Caminos, Canales y Puertos, Universidad Politécnica de Madrid, c/Profesor Aranguren, s/n, 28040 Madrid, Spain

**Keywords:** waste valorisation, concrete block, marble dust, fly ash, rice husk ash, mineral additions

## Abstract

This paper proposes binary and ternary combinations of sewage sludge ash (SSA) with fly ash (FA), marble dust (MD) and rice husk ash (RHA) as partial replacements of Portland cement in concretes with a similar dosage to that used in precast blocks, with very dry consistency. Several physical-mechanical tests were carried out on concrete specimens with curing ages of 28 and 90 days: density, water absorption, capillary water absorption, ultrasonic pulse velocity and compressive strength. The combinations of residues significantly improve the properties of the cementitious systems: 30% replacement of Portland cement provides strength values similar to the reference sample, showing the synergetic effects of the combination of the mineral additions. The significance of this research relies on the combined use of the mineral additions as well as the use of them for the precast block industry. The results show synergies among the additions and even that some of them showed relevant improvements when they are used in combination, performing better than when used individually.

## 1. Introduction

The management of the unavoidable waste generation in modern societies has become one of the most serious environmental challenges. Thus, negligent or improper waste management produces a substantial effect on the receiving environments and can cause contamination in the water, ground, and air, and contributes to climate change, affecting ecosystems and human health. However, appropriate waste management becomes a resource that contributes to the reduction in raw material consumption, produces remarkable benefits for the preservation of natural assets, reduces the impact on climate change and boosts sustainable development [1].

The construction field, encompassing civil works and the building industry, is responsible for 60% of the raw materials extracted from the lithosphere [2]. The production, transportation, and assembly of the most widely used construction materials such as steel and concrete also involves large amounts of energy. In addition, the demolition debris gathered with construction waste is also significant, totaling around 40 million tons per year in Spain alone [3]. Such figures show the massive potential benefits that the use of waste generated by industrial activities in construction works could imply.

This paper is a feasibility study for the use of four waste products generated from industrial processes to be applied as mineral additions for cement in concrete. Moreover, three of them originate from outside the construction activity. These were sewage sludge ash (SSA) coming from wastewater treatment plants, fly ash (FA) generated from thermal coal plants, marble dust (MD) derived from the stone cutting industry and rice husk ash (RHA) from rice processing plants. Mineral additions are added to cementitious materials such as concrete or Portland cement-based mortars in order to achieve additional properties or improve other features, especially in terms of durability or mechanical properties [4]. The additions can be inorganic but can also play active roles with pozzolanic materials or other with latent hydraulic properties. The pozzolanic reaction is achieved by adding the aluminosilicate residue as supplemental cementing materials (SCM) in ordinary Portland cement (OPC). The use of SCM is a viable way of achieving value-added utilization of waste in the cement industry [5].

Research dealing with the use of mineral admixtures began in the 1950s, mainly concentrated on their possible use in the production of Portland cement, mortars or special concrete types. At the time of writing, there has been an exponential growth in the use of all types of mineral additions, with global production estimated to have been as follows (in million t/year): 370 FA, 35 blast furnace slag, 20 RHA and 2 of silica fume [6].

Two of these residues, SSA and MD, represent a serious environmental problem at the local level due to the volume produced and gathered. Regarding SSA, around 1.06 million tons of dry material are produced annually in Spain [7]. This type of residue can be treated to be used as a fertilizer (65 to 80%), controlled landfill (8 to 20%) or, in some cases, is incinerated in order to reduce its volume (4 to 10%, although the trend is to increase the percentage up to 20 or 25% as an average value in Europe) [7,8,9]. Given that these residues have a heavy metal composition after incineration, turning them into a potential pollutant, there is intensive interest in finding alternatives for using them as a landfill [10].

Regarding MD, it is worth noting that it is not a contaminant waste, being composed of 98% calcium carbonate. The main impacts of such an industry remain at the local scale. In the case of Spain, Alicante province produces 70% of the domestic marble, with Spain being one of the most relevant producers in the world (second in Europe and seventh in global production). This industry produces approximately 500,000 sludge tons from polishing and cutting natural stones [11].

The effect of applying these wastes—either individually or combined—for the partial substitution of cement in conventional matrixes and binders is well known:

- As an example, in the case of SSA, it has been shown to be adequate to partially replace cement up to proportions of 10%, or with 2% substitution of the mix sand. In such cases, the specimens showed analogous compressive strengths compared with control specimens. Regarding this leaching study, results indicate that the mixture of mortar or concrete with ash, cement and sand induced the stabilization of Mo and Se, so in this way, this constitutes an effective treatment of the ashes [12]. Previously published research revealed the good mechanical properties of mortars with SSA [13,14]. These enhancements have been attributed to the pozzolanic activity of SSA [9,15,16]. Nevertheless, it should also be mentioned that other research focused on reused materials has shown that the pozzolanic activity of ashes should be considered at least as weak when it is compared with other widely known pozzolanic materials [17]. Moreover, the irregular shapes of the particles that limit their performance as a solid lubricant result in a reduction in the workability of the mixes, as has been concluded in references [18,19].
- The use of FA as a mineral admixture has been widely known for more than three decades. The most significant properties rely on its pozzolanic activity for the medium and long term, with spherical particles and high amounts of vitreous SiO_2_ and Al_2_O_3_ [20]. In addition, incorporating FA in Portland cement composites has been shown to improve the workability and consistency of concrete and mortar matrixes [21].
- SSA has been shown to possess significant pozzolanic activity for ternary combinations for the production of cement/SSA/FA binders. This pozzolanic activity increased the mechanical properties of mortars in the age time-period from 7 to 28 days. In these ternary combinations, SSA harmed the fluidity of the mixes while the addition of FA enhanced it [22,23].
- Published research has shown that MD in cementitious composite materials, employed as an addition to substitute up to 10% of sand, enhances the cohesion of the mixture, maintaining the compressive strength, reducing permeability, and even improving the mechanical performance in comparison with the effect of the same amount of limestone filler [24,25]. Regarding the properties of self-compacting concrete by adding MD, the results have been shown to meet the requirements in the standards. Given that with the addition of fine particles with good properties, a greater compactness is achieved, thus improving the mechanical performance [24,26]. The plastic viscosity of the mixtures with the addition of MD tends to increase with increasing content rates and has been rectified by using superplasticizers.
- RHA has demonstrated a significant positive impact on both the mechanical and durability properties in cementitious matrices [27,28]. Research dealing with RHA, mainly substituting part of the cement in mortars and concrete, has revealed that when replacing up to 25% of the cement, the results obtained were at least the same compared with a reference conventional concrete [29]. When the percentage of substitution was increased up to 30%, durability and consistency remained, showing improvements. In such cases, the compressive strength only showed improvements for older ages [30].

It is also worth summarizing the recent advances as well as the limitations of the SCM used in this paper [31]. SSA was not only considered as SCM in mixed cements but also for a great scale of construction and building materials such as paving stones, roof tiles, bricks, or the production of lightweight aggregates. When SSA was used as a partial substitute for cement, a reduction in compressive strength was found. Hence, the limitations in the use of both SSA and SCM were noticed. MD has been shown to be a suitable SCM for self-compacting concrete. Nevertheless, there is still a lack of studies to assess the properties of mortars or concrete with MD. Concerning RHA, its use as a substitute for cement in concrete has shown to be viable. However, the fineness can affect the compressive strength for greater particle sizes and small specific surface areas. As regards FA, it improves durability, density, and concrete workability. FA diminishes concrete water demand, porosity, and permeability.

A previous work about concrete blocks, where the fine aggregate was substituted by fly ash and the coarse aggregate was substituted by bottom ash, is relevant to the specific application analyzed in this paper, namely concrete for block manufacturing. The results show that they provide fire passive protection and that no loss was found in terms of the mechanical properties of the product. Furthermore, according with the results of the leaching tests, there was no evidence of environmental problems [32]. At the time of writing, there is a lack of research concerning the application of the types of waste proposed in this study in order to produce concrete blocks. Nevertheless, several works dealing with the replacement of aggregates by debris or construction and demolition waste for precast blocks have been published [33,34]. Some other examples of the synergy between cement and concrete industry such as the use of waste glass and fly ash to produce geopolymer cement have been published recently [35,36]. In the references, it can be seen that when blocks were manufactured with 100% substitution with recycled aggregates, the blocks produced with natural aggregates were stronger. However, 85% of the specimens met the requirements of the standards, which revealed the feasibility of the use of these wastes for structural concrete. The remaining 15% of the specimens were only suitable for non-structural applications.

This paper reports on the effect of combining SSA with diverse mineral residues on the properties of concrete to be used—with characteristics in accordance with the manufacturing process for precast block production. The significance of this research relies on the replacement of Portland cement by several materials proceeding from waste that will expand knowledge about future possibilities to improve the properties of cementitious composites in both fresh and hardened states. Moreover, the waste materials were used individually and combined in binary and ternary combinations. The synergies among the mentioned wastes, considering with special care SSA, were assessed and will boost the future exploitation of them. It is worth noting that this study is the continuation of previous works published by the authors related to the replacement of cement mortars and concretes by binary and ternary combinations of waste materials [37].

This paper shows the results of several concrete cubic specimens with different sizes, configurations and manufacturing processes compared with the concrete blocks produced in a plant. That is to say, the results are not directly applicable to the blocks, although the same mix proportioning as the one that would be used in the regular concrete block manufacturing in the plant was used. Having said that, this study could serve as the initial phase for the production of pilot precast blocks with the use of the mineral additions that have been shown to yield the most effective results in this initial trial stage. This issue is covered in more detail in the experimental section.

The CE mark for construction and building materials is well known given that it is required in order to commercialize any product. In order to obtain this quality label, manufacturers have to establish and guarantee certain characteristics of their products although it does not refer to compliance with any explicit and minimal requirements. That is to say, some of the additions studied could be considered unsuitable in terms of quality for certain uses, while their qualities could be perfectly suitable for other uses.

The significance of this research relies on the combined use of the mineral additions as well as the use of them for the precast block industry. In order to do so, compressive strength, water absorption and density of concrete mixtures were assessed in order to meet the requirements for the CE marking of the blocks. In addition, this study was focused in the binary and ternary combination of such mineral residues, assessing not only the results but also the synergetic effects that appear. Indeed, certain materials showed that they improve their properties when they are used in combination, performing better than when used individually.

## 2. Experimental Campaign

### 2.1. Materials

In this study, the following mineral additions were used: SSA, FA, MD and RHA. SSA was obtained from the wastewater treatment plant of Pinedo (Valencia, Spain). The product was supplied in bulk and was obtained from the release of a fluidized bed incinerator, subjected to a maximum temperature of 800 °C. The FA was also supplied in bulk and came from the coal power plant of Andorra-Teruel (Teruel, Spain). Regarding MD, it came from a refuse dump in Novelda (Alicante, Spain) that accumulates the waste from various local industries. RHA originated in an energy recovery plant that uses rice husk for fuel. The product was ground to reduce particle size and internal porosity before it was supplied by the company DACSA, (Valencia, Spain).

Spanish Portland cement type CEM II/B-M (S-LL)-42.5R (supplied by Cemex) employed in this study was chosen in order to be the same used for the production of the blocks in the plant. This cement type is a blended cement of 42.5 N/mm^2^ high early strength class. The composition was in accordance with Spanish Standard RC-16 (by mass): (a) clinker, 65–79%; (b) a mix of blast furnace slag and limestone, 21–35%; (c) 0–5% of other minor components. Concerning the aggregates, they were two crushed limestone aggregates: 0–4 mm size (F-0/4) and 2–8 mm size (F-2/8). These aggregates were supplied by the blocks manufacturing plant.

### 2.2. Mix Proportioning

The control specimen was manufactured with an analogous mix proportioning of the concrete blocks produced in the plant. The consistency was dry with null slump in the test. This is a key-point in the manufacturing process of the precast blocks given that the concrete is poured into the molds for a very short time-period before it is removed for curing.

Table 1 summarizes the dosages used in the different mixtures.

It can be observed that the predominant addition is SSA, as it appears in almost all combinations. The control specimen (C) is accompanied by the following samples: substitutions of 10, 20 and 30% of cement by SSA (identified by references 10, 20 and 30, respectively); 20% substitution of cement by the binary combination (10 + 10%) of two residues: SSA + FA, SSA + MD and MD + FA (identified as 21, 22 and 23, respectively); and cement replacement by 30% of residue combinations: mix 31 (10%SSA + 10%FA + 10%RHA) and mix 32 (20%SSA + 10%RHA).

In order to assess the variations of the properties, the weight of binder was kept constant as the cement plus the mineral addition. Therefore, the water/binder ratio was 0.68 in all the formulations. If Table 1 is observed in more detail, the values of water and cement were lower than the conventional for concrete mix design. Moreover, the high proportions of fine aggregates also entailed higher water absorptions. This mix proportion produced concrete with a very dry consistency in the fresh state, which was a requirement for this specific application.

### 2.3. Experimental Program

The raw materials were characterized by means of a particle and size distribution of the aggregates and sand equivalent test. In addition, X-ray fluorescence analysis (XRF) and scanning electron microscopy (SEM) of the mineral additions used in the study (SSA, FA, MD and RHA) were performed.

For every concrete type, six specimens were cast: three were used for mechanical testing and the other three for physical tests. Thus, with these specimens, the following test program was conducted: dry mass density (*Dmd*), water absorption (*Abs*), capillary water absorption (*Cap*), ultrasonic pulse velocity (*Upv*) and compressive strength (*Cs*). This test program was designed in accordance with the standard EN 771-3 Specification for masonry units—Part 3: Aggregate concrete masonry units (Dense and lightweight aggregates), applied for precast concrete blocks. In the standard, all the previously mentioned tests (except *Upv*) are listed in the initial tests. SEM was also used on the concrete specimens.

### 2.4. Procedure

#### 2.4.1. Previous and Complementary Tests

The particle size and distribution was assessed according to the standards UNE-EN 933-1 and UNE-EN 933-1/A1. The aggregate sizes were separated by means 9 sieves with openings in the range from 63 mm to 63 µm. A sand equivalent test (SE) following the standard UNE-EN 933-8 was completed for the fraction 0/2 mm.

The X-ray fluorescence technique (XRF) was performed with a sequential rhodium tube and beryllium window X-ray spectrometer (Philips Magix Pro, Amsterdam, The Netherlands). This technique was applied in order to achieve some additional data from the chemical composition of the mineral additions that could enhance the comprehension and discussion of the results.

Regarding the scanning electron microscope (SEM), it was performed by a JEOL JSM-840 equipment, Tokyo, Japan.

In the XRD technique, applied for the qualitative identification of the mineralogical composition of the concrete samples, a JSO-Debyeflex Seifert 2002 (Alicante, Spain) equipment was used, provided with a copper cathode and nickel filter.

#### 2.4.2. Manufacture of the Concrete Specimens

The aim of this study was to reproduce as much as possible the concrete mix design and pouring procedures used in the real industry. Thus, the sequence and procedures followed by the local plant for the manufacture of the blocks were carefully studied before any batch was performed in the laboratory. These procedures were assumed as a reference for the manufacture of the blocks: concrete was poured into the molds and a single blow of compaction-vibration was carried out; then, the molds were removed and the blocks were transported to the curing chamber.

The reproduction of the block industrial plant process at the lab involved the handling of concrete with a very dry consistency. This condition implied a highly complex management and handling of the specimens at the lab. Therefore, the standard UNE-EN 12390-2 was taken as starting point which led to the following procedures:

- Cubic specimens of dimensions 150 × 150 × 150 mm^3^ were manufactured by pouring the concrete on PVC molds following the standard UNE-EN 12390-1. Before the molds were filled, a release agent was sprayed on the walls and they were protected with plastic sheets in order to ease the specimen removal. This operation allowed for extracting the concrete element despite the dry consistency of the mixture.
- In order to perform an adequate mix of the components, the humidity was carefully controlled. This is of great importance given the strict consistency requirement of the mix design. Some of the components such as cement and the mineral additions were dry in origin. In the first step of the mix sequence, those dry components were mixed in absence of water. Then, the mixing of aggregates and water was conducted. The concrete was poured into the mold and it was compacted for 30 s at low speed by means of a pneumatic hammer (Milwaukee Kango-900). The specimens were stored in a moisture room for 24 h and were demolded by means of compressed air. After this, the specimens were stored in a curing chamber at 90% RH 20 °C until the testing age. In specimen “30” two forms of curing were used (the one called simply as “30”, cured in a humidity room, and the so-called “30’ ” cured under water.

#### 2.4.3. Tests on Concrete Specimens

The density and absorption were assessed following the standard UNE-EN 12390-7. Regarding the water absorption, it was carried out in agreement with the standard UNE-83982, measuring at intervals of 5, 10, 15, 30, 60, 120, 180 and 240 min for 4 h. Equation (1) was used to compute the capillary absorption (*Cap*), Equation (1):

(1) $$Cap=\frac{\left(Q_{f}-Q_{0}\right)*10}{A*\sqrt{t}}$$

where *Cap* is given in kg/m^2^min^0.5^, *Q_f_* is the mass after 4 h testing (g), *Q*_0_ is the initial mass (g) before starting the test (the samples were previously dried to constant mass in an oven), A is the section of the specimen (225 cm^2^), and *t* is the duration of the test (240 min).

Equation (2) was used to determine the mass increase (*Q*) respect to the initial mass:

(2) Q= 100×(QtQ0−1)

where *Q* is given in %, *Q_t_* is the final mass (g) after the *t* test time and *Q*_0_ is the mass (g) before the test starts (specimen dried in an oven to constant mass and cooled at room temperature into a desiccator).

According to the theoretical model formulated by Fagerlund [38], with respect to the average mass gain by capillary water absorption (*Q*) of concrete samples versus time, during state 1, water filling would occur by absorption through the capillary pores of specimens. When the time corresponding to $\sqrt{t_{n}}$ is reached (state 2), the water would arrive at the upper surface of the specimen, and there will be a continuity of the capillary filling through the pores of air by the process of diffusion and dissolution of air.

On three specimens of each concrete type, the *Upv* tests were performed by means of a Steinkamp BP-5 instrument and following the standard UNE-EN 12504-4. After the *Upv* test, compressive strength tests were carried out on the specimens. The direct measurement method was applied: transducers were placed on the transverse axis, in the direction in which the concrete was poured.

The compressive strength tests were completed on a Suzpecar CMP-150 t press, according to UNE-EN 12390-3, being the load applied in the same direction at which the concrete was poured when filling the molds.

#### 2.4.4. Statistical Data Analysis

In order to perform the statistical analysis, the software Statgraphics was used.

## 3. Results and Discussion

### 3.1. Previous Tests

#### 3.1.1. Characterization of Aggregates

Table 2 shows the distribution of the aggregates on each of the sieves.

Concerning the Sand Equivalent Index, the results obtained show that the fine aggregate met the requirement of the Spanish Structural Code EHE-08 for concrete elements under general environmental exposures such as class I, IIa or IIb and not subjected to other explicit type of exposure. The requirement is to be equal or higher than 70% and the result of SE test of the fine aggregate was 68.4%.

#### 3.1.2. X-ray Fluorescence Analysis on Mineral Additions

**Figure 1:**
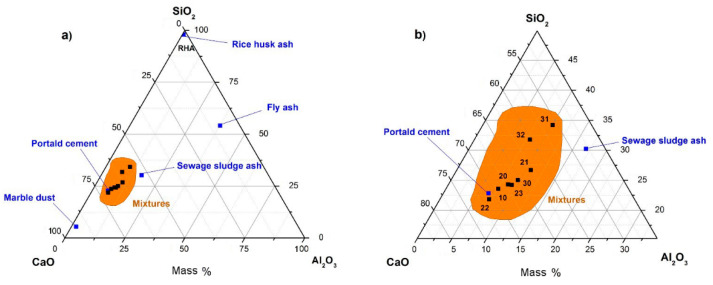
Elemental compositions of the materials used in the study on the SiO_2_-CaO-Al_2_O_3_ ternary diagram (based on mass%). (**a**) includes all materials; (**b**) detail of the mixtures, with the following references: 10 (substitution of 10% of the cement by sewage sludge ash (SSA)), 20 (20% SSA), 21 (20% SSA/fly ash (FA)), 22 (20% SSA/marble dust (MD)), 23 (20% MD/FA), 30 (30% SSA), 31 (30% SSA/FA/rice husk ash (RHA)).

The X-ray fluorescence (*XRF*) results are shown in Table 3 and Figure 1.

SSA presented a significant content of CaO (30.24%), SiO_2_ (17.27%) and Al_2_O_3_ (9.64%). With this composition, SSA is expected to behave as an active mineral addition for concrete. It merits mentioning that the amounts of SO_3_, P_2_O_5_ and Fe_2_O_3_ were also remarkable. In the case of FA, the amounts of silica (36.70%) and alumina (25.57%) were much higher than in the previous case of SSA. Therefore, it could be considered as a pozzolanic mineral addition that could be classified in class F Fly Ash following the ASTM C618. Regarding MD, 64.25% of the CaO was found to be calcium carbonate (CaCO_3_). Thus, the composition of MD showed that it was likely to behave as an inert mineral addition. For RHA, it can be seen that it is essentially SiO_2_, exceeding 80% of the total composition of the addition. Subsequently, RHA is highly expected to behave as a pozzolanic addition.

#### 3.1.3. Scanning Electron Microscopy (SEM) of the Mineral Additions

**Figure 2:**
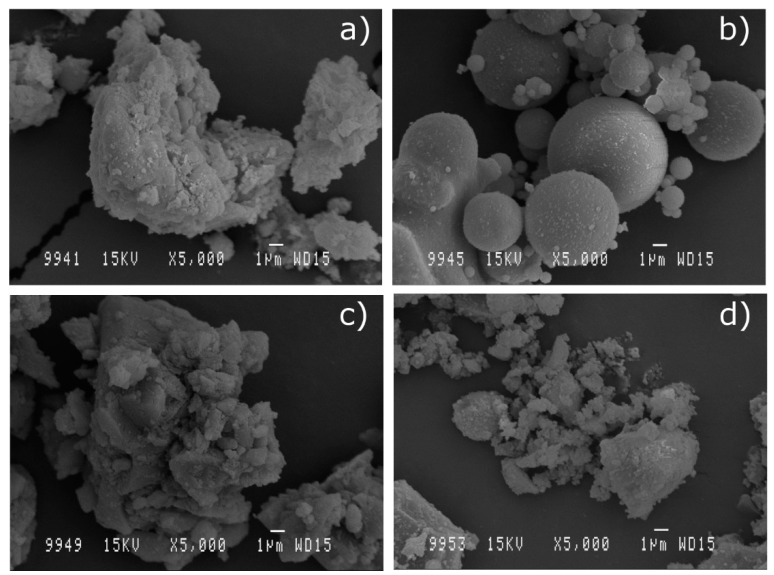
SEM micrographs (×5000) of particles of: (**a**) SSA; (**b**) FA; (**c**) MD and (**d**) RHA.

Micrographs (Figure 2) show the images obtained by scanning electron microscopy technique at magnifications of 5000, which provide information about the morphology and particles size of the residues used.

Figure 2a shows the irregular shape of particles of SSA, apparently agglomerated, with diameters mainly below 10 microns. It is worth mentioning the apparent rough texture of the particles, which could result in important water absorption phenomena, both during mixing and curing phases, as well as in hardened concrete. Figure 2b highlights the sphericity of FA particles with variable diameters not exceeding 15 microns. Its shape and dimensions give this addition the known character of solid lubricant, which facilitates the mixing of mixtures by providing low water absorption, more fluidity, and more compaction. It is noticed in Figure 2c that MD particles appear to have a more regular and less rough shape than SSA particles, with higher average size. These features will probably result in less water absorption during the different stages of the manufacture of mixtures with SSA. In Figure 2d, RHA particles are observed.

### 3.2. Results in Concrete

#### 3.2.1. Density and Water Absorption

The average values of absorption (*Abs*) and dry mass density (*Dmd*) of the mixtures cured for 28 and 90 days are shown in Table 4.

There is no large difference between relative values of densities, ranging between 93% and 100% as compared to the reference sample in mixtures cured for 90 days.

In general terms, *Dmd* values for concrete containing mineral additions were similar to plain concrete: for 28 days, the relative *Dmd* values range between 96.7 and 103.5% and 93.2 and 100.1%, respectively. Replacement of 10% yielded the highest density value, whereas 30% cement replaced concretes yielded the lowest values. This behavior suggests that the development of hydration products from mineral additions did not compensate the reduction in hydrates from cement. Additionally, combination of the two less water demanding mineral additions (FA and MD) produced denser concrete: probably, the water demand of SSA and RHA produced lower concrete compaction.

**Figure 3:**
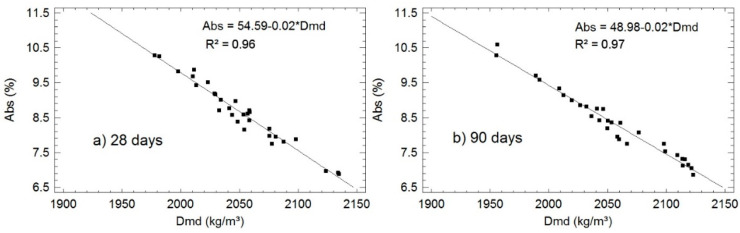
Adjusted models of correspondences between *Dmd* and *Abs* for specimens cured for: (**a**) 28 days; and (**b**) 90 days.

With the results obtained from water absorption test, the opposite tendency marked by the density is met (although with greater relative increases), which means that higher absorption values coincide with lower densities. For example, the group conformed by reference sample and samples “10”, “20” and “30”, with SSA, for higher percentage of residue, higher absorption is recorded, with an increase in relative value of up to 42% (in sample “30”), compared to reference sample, cured for 90 days. Based on Table 8 (correlation matrix) a statistically significant relationship between *Abs* and *Dmd* can be established, and more precisely, a linear, very strong relationship between the variables analyzed, with R^2^ equal to 0.96 for 28 and 0.97 for 90 days of curing. Figure 3 shows the models adjusted by linear regression in which the absorption decreases when dry mass density increases.

#### 3.2.2. Capillary Water Absorption

Table 5 shows the capillary water absorption (*Cap*) average values for samples cured for 28 and 90 days, obtained after four hours of testing.

When analyzing the results of reference sample and the three samples containing only SSA as a mineral admixture (10, 20 and 30), we noticed an increase in *Cap* by increasing the amount of cement replaced by SSA, with significant increases observed between these mixtures, reaching a maximum of 371.4% compared to the control specimen cured for 90 days.

Regarding the increases obtained between the two curing ages of each sample (*Δ* in Table 5), the highest decrease in the value of *Cap* occurred in reference sample (−34.4%); a remarkable decrease also occurs, but to a lesser extent, in sample 10 (10% SSA) and those containing FA. Apparently, the flattering effect of *Cap* caused by SSA in small quantities, as in sample “10”, happens under the age of 90 days, and for additions in larger amounts but combined with FA, this effect is cancelled to some extent.

In binary combinations of additions, considering sample “20” (20% SSA) as a reference, the value resulting from *Cap* in sample “21” (SSA + FA) was similar (0.83 versus 0.84 kg/m^2^min0.5 at 90 days). Lower values compared to reference sample were found in Samples “22” (SSA + MD) and “23” (MD + FA). For all the mixtures studied, Sample “23” showed the closest value to reference sample, with a relative value of 124.4% at 90 days. This result is in line with what was observed in the previous section, where sample “23” also showed the nearest values regarding to density (99.4%) and absorption (105.6%) for combinations of mineral admixtures after 90 days of curing. For ternary combinations of additions at 90 days, lower *Cap* values than those achieved by sample “30” (30% SSA) are obtained, but in the best case (Sample “31”) it is nearly three times the one found for the reference sample (280.5%).

**Figure 4:**
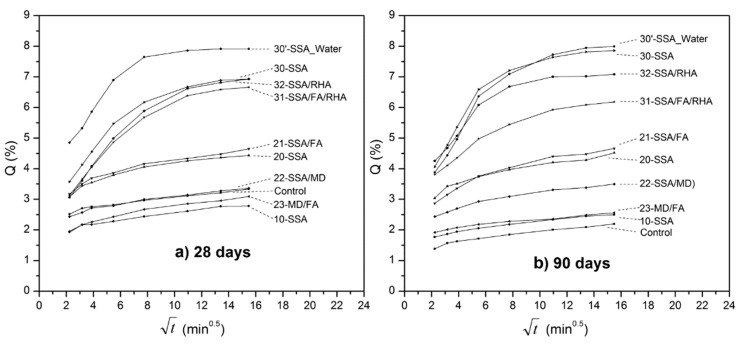
Mass gain (average) by capillary water absorption *(Q)* against square root of time for specimens cured: (**a**) 28 days; (**b**) 90 days.

The behavior regarding the mass gain average by capillary absorption *(Q)* during the four-hour test is shown in Figure 4.

Two zones are observed:

1. In the bottom of the graphs, therefore with lower values of capillary absorption, including the reference sample, samples “10” and “20”, and the binary combinations of mineral admixtures (“21”, “22” and “23”). These mixtures show lines parallel to each other, which indicates similar behavior during the test. After four hours of testing, the upslope remains, which means that water do not reach the upper surface in any of these samples, so that the filling is produced by water absorption through the capillary pores (state 1 of the Fagerlund model);
2. Upper half of the graphics, therefore with higher capillary absorption values, including samples “30” and “30’ ” (cured under water), and the ternary combinations of mineral additions (“31” and “32”). A similar behavior between the specimens of this series is observed (parallel lines), but the main novelty of the parameters analyzed so far is that the slope is very low when the test is finished, which means that the water front reaches the top of the specimens, saturating the specimens faster. The time t_n_, which would correspond to the continuity of the capillary refill through pores of air by the process of diffusion and dissolution of air, will be approximately 120 min in sample “32” and between 180–240 min in samples “30” and “30’ ” (specimens cured for 90 days).

**Figure 5:**
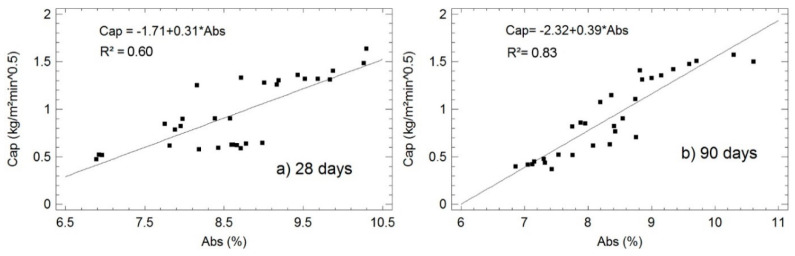
Adjusted models of the relationship between *Cap* and *Abs* in specimens cured for 28 (**a**) and 90 days (**b**).

Based on the correlation matrix and statistical study shown in Table 8, it is possible to conclude that there exists statistical correlation between *Cap* and *Abs*. By examining Figure 5, it can be better understood that the relationships seem to be linear.

This linear fitting of the 28-day curing specimens could be classified as moderate-strong (R^2^ = 0.60). For the 90-day curing samples, the correlation coefficient increased up to 0.83 and, therefore, improving the classification to very strong. Figure 5 shows the models adjusted by linear regression.

#### 3.2.3. Compressive Strength

The compressive strength *(Cs)* of the blocks for 28 and 90 day curing ages are shown in Table 6.

As can be observed, the compressive strengths of this type of concrete was low given the high porosity of the concrete. The results of the control specimens, used as reference, were for the 28-day compressive strength equal to 7 MPa, while it reached 9.7 MPa after 90 days. Furthermore, this increment of compressive strength with the curing time was observed for all the concrete mixtures. For example, in samples *30* and *30’*, this increase is of approximately 60%, but the values of *Cs* at 28 days are the lowest of all the mixtures tested.

Samples with replacement of cement by *SSA* show a decrease in *Cs* compared to the reference sample with the same curing age: sample “30” (90 days) does not exceed the 77% of the reference. This behavior can be related to the low pozzolanic reactivity of this mineral admixture. Sample “30’” suffers a significant drop compared to its counterpart, 5.6 vs. 7.4 MPa, possibly due to the washing effect of a porous matrix, produced during its curing under water.

Combinations of waste improve the results of the simple additions of *SSA* in all cases, with values close or equal to reference sample in several mixtures cured for 90 days: 22 (SSA + MD), 23 (MD + FA) or 32 (SSA + RHA). Sample 32 should be highlighted because, with a high replacement of cement (30%) by 20%SSA and 10%RHA, the compressive strength achieved is equal to the reference sample for the two curing ages: 7 MPa at 28 days for both cases and 9.5 versus 9.7 MPa for 90 days. Therefore, a significant saving of Portland cement is achieved with excellent mechanical response. This behavior in terms of mechanical strength may be attributed to the high reactivity of RHA. This mineral admixture usually presents a good pozzolanic reaction rate, and the chemical reaction of portlandite and reactive silica from RHA produces an extra quantity of calcium silicate hydrate (C-S-H). Mixtures containing MD also showed high compressive strength values: this can be related to the ability of carbonates to chemically react towards reactive alumina (present in SSA or in FA). This reaction produces cementing calcium carboaluminate hydrates [39].

The relative *Upv* ranged between 95 and 100% of the reference sample. According to the correlation matrix (Table 8), it can be observed that there is no statistically significant relationship between *Cs* and *Upv*, since the *p*-value in the ANOVA table is greater than 0.05 (0.80). The correlation coefficient (R^2^) is equal to 0.01, which indicates a very weak correlation between variables. This behavior may be due to the high porosity of the matrix studied, making that Upv technique is not appropriate for analyzing the development of compressive strength.

**Figure 6:**
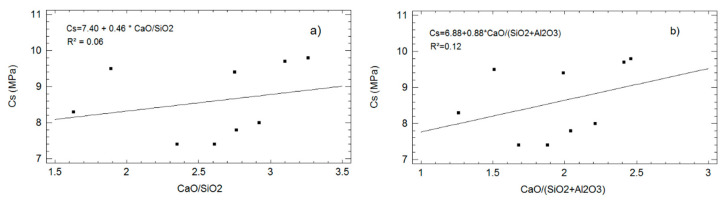
Adjusted models of the relationship between *Cs (90 days)* and two parameters: (**a**) CaO/SiO_2_; (**b**) CaO/(SiO_2_ + Al_2_O_3_).

Figure 6 shows the adjusted models through linear regression among *Cs* and two parameters: CaO/SiO_2_ and CaO/(SiO_2_ + Al_2_O_3_). With the available data, no clear correlation appears.

This might occur because in addition to the reactivity of the mineral additions, the degree of compaction (density) is also an influencing factor. Moreover, it should be considered that the reactivity of the additions is different. In some cases, there was a large amount of reactive silica available (RHA). In some others, there was a combination of silica and alumina (FA). In both cases, RHA and FA, there is a significant pozzolanic contribution, much higher than the case of SSA. In the case of MD, there was a large quantity of carbonates that can ease the development of mechanical strengths by the formation of calcium carboaluminates hydrates. This was found for recent studies dealing with LC3 [39,40].

#### 3.2.4. Scanning Electron Microscopy (SEM) of Concrete

The following images were obtained by the scanning electron microscopy (SEM) technique on concrete samples cured for 90 days.

**Figure 7:**
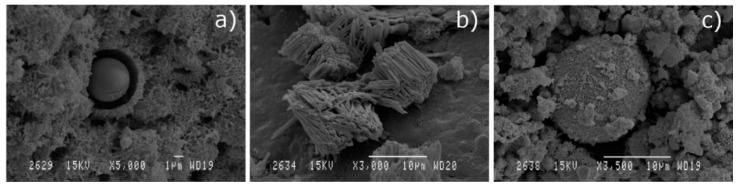
SEM micrographs of concrete samples (**a**) 21-*SSA*/*FA*, (**b**) 22-*SSA*/*MD* and (**c**) 23- *MD*/*FA*, cured for 90 days.

Figure 7 shows some images of concrete with replacing percentage of 20%.

In micrographs Figure 7a,c, some fly ash particles are surrounded by hydration compounds, and in Figure 7b, some agglomerated needles of ettringite are depicted.

**Figure 8:**
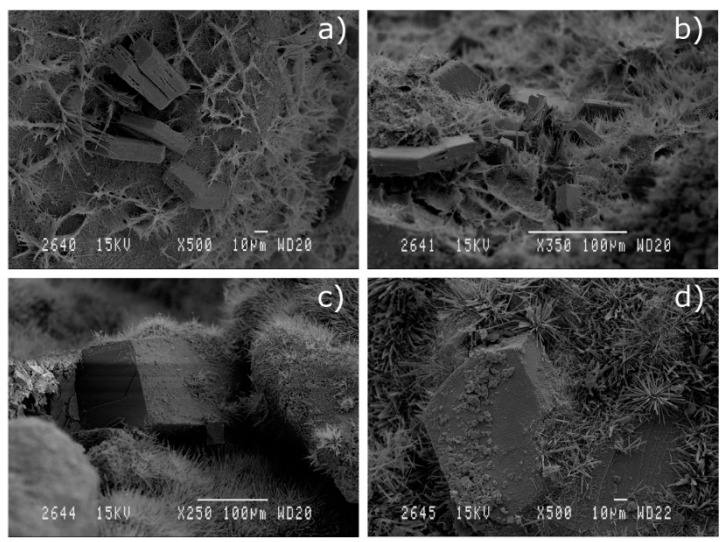
SEM micrographs of concrete samples 30’-*SSA*_water (**a**) and (**b**) and 30-*SSA* (**c**,**d**), cured for 90 days.

In Figure 8, micrographs for 30%-SSA replaced concrete are shown.

The presence of portlandite and fibrillar C-S-H is observed in Figure 8a,b. Large crystals of calcium carbonate (probably calcite, produced by carbonation of portlandite) and needle-shaped ettringite are identified in Figure 8c,d.

**Figure 9:**
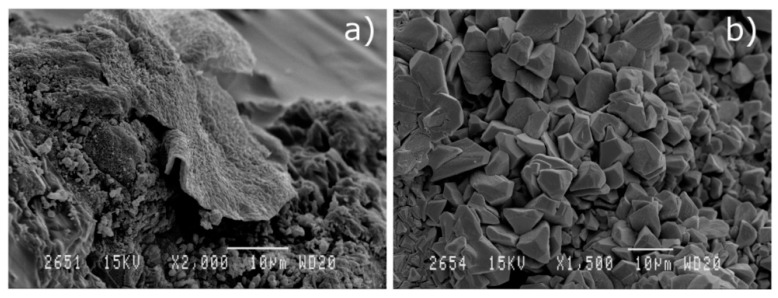
SEM micrographs of the concrete samples: (**a**) 31-*SSA*/*FA*/*RHA* and (**b**) 32-*SSA*/*RHA*, cured for 90 days.

In Figure 9, some micrographs of 30% replaced concrete with two or three mineral additions are shown.

It is possible to see in Figure 9a what might be the outer side of a portion of rice husk in concrete sample 31-*SSA*/*FA*/*RHA*, with a rough texture. The crystal formation shown in Figure 9b must be highlighted, due to intense carbonation in concrete sample 32-*SSA*/*RHA*.

#### 3.2.5. Correlation Matrix

A multivariate statistical analysis is summarized in Table 7 and Table 8. It shows the data for all the representative specimens of the samples analyzed for both curing ages (28 and 90 days).

The measurements of bias (degree of symmetry) and kurtosis (degree of sharpness or flatness), which can be used to determine if the sample comes from a normal distribution (bell curve), are of particular interest. A variable (*Cs*-28 days of curing age) with a value of bias (−2.1) in the limit of the range of −2 to +2, can be observed, which indicates significant deviations from normality. By extending the former analysis, it is observed that this atypical value is due to the low values of *Cs* achieved by sample “30′ ”. However, since the *p*-value for the Grubbs test is greater than 0.05 (0.41), it can be listed as a non-significant value with a significance level of 5.0%, assuming that the others values follow a normal distribution, so it was decided to be included in the correlation matrix (Table 8). This anomaly can be justified by the fact that sample “30′ ” was subjected to a different curing process: as discussed in previous sections, its curing process under water caused a wash effect of the cement for being a very porous concrete.

A matrix of correlations among all the variables studied is shown in Table 8. The table encompasses the following information:

(a) The Spearman correlations between each variable pair are shown at the top, on the diagonal matrix. These values, ranking between +1 and −1, measure the strength of the linear relationship between the variables. At the bottom of the cell, the *p*-value shown is derived from the analysis of variance (ANOVA). ANOVA tests the statistical significance of the estimated correlations (for *p*-value < 0.05, the correlations are said to be significantly different from zero, with a reliability of 95%);
(b) The determination coefficients R^2^ between variables are located below the diagonal matrix. These variables are colored according to the scale of Evans [41], (1–0.8 very strong, 0.8–0.6 strong, 0.6–0.4 moderate, 0.4–0.2 weak and 0.2–0 very weak). This matrix makes clear the strong relationship among capillarity, absorption and. Accordingly, the analysis of the simple regression models (see Figure 4, Figure 5 and Figure 6) included in the corresponding section is completed.

## 4. Conclusions

The replacement of 30% of cement by SSA involves a decrease in density and compressive strength compared with the ones in the control specimen. Nevertheless, replacing 10% of cement by SSA or by other binary or ternary combinations of residues showed a similar response to that of the reference samples. The binary combination of 20% replacement of SSA/RHA and the ternary combination of 30% replacement of SSA/FA/RHA showed even higher compressive strength than the reference samples. These combinations could be commercially feasible. In addition, it is a valid solution for the sustainability regulations proposed by the directives of the Spanish National Plan of Sewage Sludge.

There is a direct correlation between the concrete density and the general and capillary absorption values of this type of concrete: when density decreases, both the general and the capillary absorption values increase.

## Figures and Tables

**Table 1:** Concrete mix proportioning (kg/m^3^) and dosages of mineral additions (SSA: sewage sludge ash; FA: fly ash; MD: marble dust; RHA: rice husk ash).

Reference		Aggregate Fraction		Cement	Mineral Addition								Water
		F-0/4	F-2/8		SSA		FA		MD		RHA		
0	C	1227	571	125.6	-	-	-	-	-	-	-	-	85.6
10	SSA	1227	571	113.0	12.6	10%	-	-	-	-	-	-	85.4
20	SSA	1227	571	100.4	25.1	20%	-	-	-	-	-	-	85.4
21	SSA/FA	1227	571	100.4	12.6	10%	12.6	10%	-	-	-	-	85.4
22	SSA/MD	1227	571	100.4	12.6	10%	-	-	12.6	10%	-	-	85.4
23	MD/FA	1227	571	100.4	-	-	12.6	10%	12.6	10%	-	-	85.4
30	SSA	1227	571	87.9	37.7	30%	-	-	-	-	-	-	85.4
31	SSA/FA/RHA	1227	571	87.9	12.6	10%	12.6	10%	-	-	12.6	10%	85.4
32	SSA/RHA	1227	571	87.9	25.1	20%	-	-	-	-	12.6	10%	85.4

**Table 2:** Particle size and distribution of the two aggregate fractions used (PR: partially retained; CR: cumulative Retained; F1: fraction 1; F2: fraction 2).

Sieve (mm)	PR (g)		CR (g)		% CR		% PASSING	
	F1	F2	F1	F2	F1	F2	F1	F2
63	0	0	0	0	0	0	100	100
31.5	0	0	0	0	0	0	100	100
16	0	0	0	0	0	0	100	100
8	0	18.9	0	18.9	0	1.4	100	98.6
4	0	1020.0	0	1038.9	0	78.3	100	21.7
2	84.1	187.8	84.1	1226.7	26.9	92.5	73.1	7.5
1	80.9	31.2	165.0	1257.9	52.7	94.9	47.3	5.1
0.5	47.5	13.2	212.5	1271.1	67.9	95.9	32.1	4.1
0.25	29.2	7.9	241.7	1279.0	77.2	96.5	22.8	3.5
0.125	18.0	5.7	259.7	1284.7	83.0	96.9	17.0	3.1
0.063	10.4	4.8	270.1	1289.5	86.3	97.2	13.7	2.8
Receiver	42.9	36.5	313.0	1326.0	100	100	0	0
Total (without fines)	270.1	1289.5					**Designation**	
Total (with fines)	313.0	1326.0				d	0	2
% fines	13.71	2.75				D	4	8

**Table 3:** Chemical composition (oxides, in %) for the mineral additions used.

Oxide	SSA	FA	MD	RHA	Oxide	SSA	FA	MD	RHA	Oxide	SSA	FA	MD	RHA
Na_2_O	0.94	0.17	0.39	0.09	TiO_2_	0.92	0.90	-	0.02	Rb_2_O	0.01	0.01	-	-
MgO	3.22	1.06	6.90	0.67	Cr_2_O_3_	0.17	0.03	-	-	SrO	0.25	0.11	0.04	0.01
Al_2_O_3_	9.64	25.57	1.39	0.44	MnO	0.07	0.04	-	0.16	SnO_2_	0.03	-	-	-
SiO_2_	17.27	36.70	3.77	81.57	Fe_2_O_3_	8.52	15.72	0.35	0.16	BaO	0.14	0.10	-	-
P_2_O_5_	14.25	0.65	0.09	0.95	NiO	0.03	0.02	-	-	PbO	0.04	0.01	-	-
SO_3_	8.95	1.53	1.27	0.33	CuO	0.18		-	-	Cl	0.15	-	0.13	0.28
K_2_O	1.28	1.28	0.30	3.51	ZnO	0.32	0.03	-	0.01	Y_2_O_3_	-	0.01	-	-
CaO	30.24	5.56	64.25	1.23	As_2_O_3_	0.00	0.01	-	-	ZrO_2_	-	0.02	-	-
LOI	3.38	10.47	21.12	10.57										

**Table 4:** *Dmd* and *Abs* of specimens cured for 28 (from [37]) and 90 days (*σ*: standard deviation, c*v*: coefficient of variation, *rel*: relative value compared to reference specimens with same curing age, *∆*: increase in each mixture between the two curing ages).

Reference Specimen		*Dmd*	*σ*	*cv*	*rel.*	*∆*	*Abs*	*σ*	*cv*	*rel.*	*∆*
		(kg/m^3^)	(kg/m^3^)	(%)	(%)	(%)	(%)	(%)	(%)	(%)	(%)
0	C-28	2058	1	0.0	100	-	8.7	0.1	0.6	100	-
	C-90	2115	7	0.3	100	2.8	7.1	0.3	4.0	100	−18.4
10	SSA-28	2131	6	0.3	103.5	-	6.9	0.0	0.5	80.0	-
	SSA-90	2118	3	0.1	100.1	−0.6	7.2	0.1	1.8	100.9	4.4
20	SSA-28	2085	11	0.5	101.0	-	7.7	0.2	2.5	88.0	-
	SSA-90	2062	4	0.2	97.5	−1.2	7.9	0.1	1.3	110.7	2.6
21	SSA/FA-28	2056	17	0.8	99.9	-	8.3	0.3	3.7	96.0	-
	SSA/FA-90	2043	7	0.3	96.6	−0.6	8.5	0.1	0.9	118.6	2.4
22	SSA/MD-28	2047	6	0.3	99.5	-	8.8	0.2	2.2	101.4	-
	SSA/MD-90	2060	2	0.9	97.4	0.6	8.4	0.3	4.1	117.6	−4.6
23	MD/FA-28	2074	15	0.7	100.8	-	8.1	0.3	3.8	94.0	-
	MD/FA-90	2103	9	0.4	99.4	1.4	7.5	0.2	2.9	105.6	−7.4
30	SSA-28	2010	18	0.9	97.7	-	9.7	0.2	2.3	111.7	-
	SSA-90	1972	23	1.2	93.2	−1.9	10.2	0.6	6.2	142.3	5.2
30’	SSA_water-28	1990	18	0.9	96.7	-	10.1	0.2	2.3	117.1	-
	SSA_water-90	2000	12	0.6	94.6	0.5	9.5	0.2	1.9	132.7	−5.9
31	SSA/FA/RHA-28	2039	13	0.7	99.1	-	8.7	0.5	5.8	100.2	-
	SSA/FA/RHA-90	2050	3	0.2	96.9	0.5	8.4	0.3	3.3	118.2	−3.5
32	SSA/RHA-28	2025	11	0.5	98.4	-	9.2	0.2	2.3	106.3	-
	SSA/RHA-90	2019	10	0.5	95.5	−0.3	9.0	0.2	2.3	126.1	2.2

**Table 5:** *Cap* after four-hour test in specimens cured for 28 and 90 days (σ: standard deviation, *cv*: coefficient of variation, *rel*: relative value compared to control specimen with same curing age, *∆*: increase in each mixture between the two curing ages).

Reference Specimen		*Cap*	*σ*	*cv*	*rel.*	*∆*
		(kg/m^2^min^0.5^)	(kg/m^2^min^0.5^)	(%)	(%)	(%)
0	C-28	0.61	0.02	3.1	100	-
	C-90	0.40	0.04	9.9	100	−34.4
10	SSA-28	0.51	0.03	5.4	82.5	-
	SSA-90	0.45	0.03	6.7	112.5	−11.8
20	SSA-28	0.82	0.03	3.8	134.0	-
	SSA-90	0.84	0.04	4.8	210.8	2.4
21	SSA/FA-28	0.90	0.00	0.1	147.5	-
	SSA/FA-90	0.83	0.07	8.3	209.7	−7.8
22	SSA/MD-28	0.64	0.01	1.6	103.9	-
	SSA/MD-90	0.65	0.05	7.4	164.8	1.56
23	MD/FA-28	0.60	0.02	3.3	97.5	-
	MD/FA-90	0.49	0.05	9.7	124.4	−18.3
30	SSA-28	1.32	0.01	0.4	214.8	-
	SSA-90	1.47	0.05	3.7	371.4	11.4
30’	SSA_water-28	1.51	0.12	7.8	246.1	-
	SSA_water-90	1.49	0.08	5.1	375.9	−1.3
31	SSA/FA/RHA-28	1.28	0.04	3.4	209.0	-
	SSA/FA/RHA-90	1.11	0.05	4.5	280.5	−13.3
32	SSA/RHA-28	1.31	0.04	3.3	214.4	-
	SSA/RHA-90	1.33	0.02	1.7	336.1	1.5

**Table 6:** *Cs* in specimens cured for 28 (from [37]) and 90 days and ultrasonic pulse velocity (*Upv*) in specimens cured for 28 and 90 days (*σ*: standard deviation, *cv*: coefficient of variation, *rel*: relative value compared to reference sample with same curing age, *∆*: increase in each mixture between the two curing ages).

Reference Specimen		*Cs*	*σ*	*cv*	*rel.*	*∆*	*Upv*	*σ*	*cv*	*rel.*
		(MPa)	(MPa)	(%)	(%)	(%)	(km/s)	(km/s)	(%)	(%)
0	C-28	7.0	0.2	2.7	100	-	-	-	-	-
	C-90	9.7	1.6	16.1	100	38.6	3.66	0.06	1.73	100
10	SSA-28	6.4	0.4	5.9	90.8	-	-	-	-	-
	SSA-90	8.0	0.3	4.0	82.3	25.0	3.62	0.08	2.22	97.8
20	SSA-28	5.2	0.1	1.9	75.0	-	-	-	-	-
	SSA-90	7.8	0.2	3.0	80.5	50.0	3.56	0.05	1.26	96.2
21	SSA/FA-28	6.6	0.5	7.2	95.5	-	-	-	-	-
	SSA/FA-90	7.4	1.6	16.1	76.3	12.1	3.53	0.08	2.14	94.9
22	SSA/MD-28	7.1	0.7	10.0	102.8	-	-	-	-	-
	SSA/MD-90	9.8	0.6	6.4	101.0	38.0	3.52	0.04	1.21	96.2
23	MD/FA-28	6.2	0.7	10.7	89.0	-	-	-	-	-
	MD/FA-90	9.4	1.6	17.5	96.8	51.6	3.60	0.05	1.34	98.4
30	SSA-28	4.6	0.5	11.8	66.8	-	-	-	-	-
	SSA-90	7.4	0.5	6.7	76.7	60.9	3.59	0.03	0.97	98.1
30’	SSA_water-28	3.4	0.5	14.7	49.3	-	-	-	-	-
	SSA_water-90	5.6	0.4	7.5	58.1	64.7	3.55	0.02	0.63	97.3
31	SSA/FA/RHA-28	6.2	0.3	4.3	89.6	-	-	-	-	-
	SSA/FA/RHA-90	8.3	0.5	5.5	85.6	33.9	3.63	0.06	1.54	100.4
32	SSA/RHA-28	7.0	0.3	4.8	101.0	-	-	-	-	-
	SSA/RHA-90	9.5	0.8	8.2	98.4	35.7	3.54	0.10	2.73	96.8

**Table 7:** Statistical summary of all the variables analyzed.

Statistical	*Dmd* 28	*Dmd* 90	*Abs* 28	*Abs* 90	*Cap* 28	*Cap* 90	*Cs* 28	*Cs* 90	*Upv* *90*
Count	30	30	30	30	30	30	30	30	30
Percentage	2052	2055	8.6	8.3	1.0	0.9	6.0	8.3	3.6
Standard deviation	39.7	47.9	0.9	1.0	0.4	0.4	1.2	1.4	0.1
Coefficient of Variation (%)	1.9	2.3	10.5	11.5	37.9	45.0	20.4	17.5	1.9
Minimum	1978	1955	6.9	6.9	0.5	0.4	3.1	5.3	3.4
Maximum	2135	2123	10.3	10.6	1.6	1.6	7.9	11.2	3.7
Range	156.6	167.5	3.4	3.7	1.2	1.2	4.8	5.9	0.3
Standardized bias	0.8	−0.7	−0.3	1.2	0.7	0.6	−2.1	0.2	0.1
Standardized Kurtosis	0.0	−0.6	−0.3	−0.2	−1.7	−1.6	0.3	0.2	−0.1
Skewness	0.3	−0.3	−0.1	0.5	0.3	0.2	−0.9	0.1	0.1

**Table 8:** Matrix of linear correlations of the series, including all the representative specimens of each sample with 28 and 90 days of curing time. On the diagonal, in each cell, the upper value indicates the Spearman correlation between variables, and the lower *p*-value of the Anova analysis; under the diagonal, the cell indicates the R^2^ coefficient coloured according to Evans scale [41].

	*Dmd* 28	*Dmd* 90	*Abs* 28	*Abs* 90	*Cap* 28	*Cap* 90	*Cs* 28	*Cs* 90	*Upv* 90
***Dmd*** **28**		0.83	−0.96	−0.85	−0.86	−0.80	0.22	0.26	0.15
		0.00	0.00	0.00	0.00	0.00	0.23	0.16	0.41
***Dmd*** **90**	0.63		−0.75	−0.99	−0.91	−0.94	0.42	0.48	0.40
			0.00	0.00	0.00	0.00	0.02	0.01	0.03
***Abs*** **28**	0.96	0.60		0.77	0.78	0.70	−0.19	−0.19	−0.12
				0.00	0.00	0.00	0.31	0.31	0.50
***Abs*** **90**	0.64	0.97	0.63		0.88	0.93	−0.37	−0.43	−0.41
					0.00	0.00	0.05	0.02	0.03
***Cap*** **28**	0.67	0.77	0.60	0.72		0.92	−0.39	−0.47	−0.18
						0.00	0.04	0.01	0.34
***Cap*** **90**	0.62	0.87	0.58	0.83	0.93		−0.53	−0.51	−0.26
							0.00	0.01	0.15
***Cs*** **28**	0.15	0.34	0.18	0.31	0.29	0.34		0.67	0.00
								0.00	1.00
***Cs*** **90**	0.08	0.28	0.08	0.25	0.23	0.25	0.57		0.05
									0.80
***Upv*** **90**	0.04	0.17	0.04	0.17	0.03	0.07	0.00	0.01	

	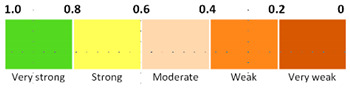								
